# Reading Ability and Mental Health: Mediating Roles of Depressive Symptoms and Behavior Problems in Chinese School-Age Children

**DOI:** 10.3390/bs15081032

**Published:** 2025-07-29

**Authors:** Xinle Yu, Kusheng Wu, Xuanzhi Zhang, Jiayu Liu, Qianfei Gu, Menghan Yu, Yanhong Huang

**Affiliations:** 1 Mental Health Center of Shantou University, Shantou 515065, China; 18xlyu@stu.edu.cn (X.Y.); xzzhang@stu.edu.cn (X.Z.); 22jyliu@stu.edu.cn (J.L.); 22qfgu@stu.edu.cn (Q.G.); 22mhyu@stu.edu.cn (M.Y.); 2 Shantou University Medical College—Faculty of Medicine of University of Manitoba Joint Laboratory of Biological Psychiatry, Shantou 515065, China; 3 Department of Preventive Medicine, Shantou University Medical College, Shantou 515041, China; kswu@stu.edu.cn; 4 School of Public Health, Shantou University, Shantou 515063, China

**Keywords:** dyslexia, reading ability, depressive symptoms, behavior problems, mediating loop

## Abstract

**Background:** Developmental dyslexia (DD) affects reading ability and exacerbates mental health challenges among children. This study examines the relationships between reading ability, depressive symptoms, and internalizing and externalizing behavior problems in Chinese school-age children, focusing on potential mediating effects. **Methods:** A case–control study was conducted with 44 dyslexic children and 81 controls from Shantou, China. Assessments included phonological processing tasks for reading ability, the Depression Self-Rating Scale for Children (DSRS) for depressive symptoms, and the Child Behavior Checklist/6–18 (CBCL/6–18) for behavior problems. Mediation analyses were performed using the PROCESS macro 4.1 for SPSS. **Results:** Dyslexic children showed significantly poorer reading ability (all phonological tasks, *p* < 0.001), higher prevalence of depressive symptoms (40.9% vs. 17.3%, *p* < 0.01), and greater behavior problems (internalizing and externalizing, both *p* < 0.001) compared to controls. Both depressive symptoms and behavior problems significantly mediated the effects of reading ability on each other, forming a feedback loop that further impairs reading skills. Externalizing behavior problems showed the strongest mediation effect, explaining up to 33.53% of the relationship between depressive symptoms and reading ability. **Conclusions:** The study reveals a complex interaction between reading ability, depressive symptoms, and internalizing and externalizing behavior problems in Chinese school-age children, suggesting the need for integrated interventions targeting educational and psychological aspects. Further longitudinal research is needed to clarify causal relationships and refine intervention strategies.

## 1. Introduction

Developmental dyslexia (DD) is a neurodevelopmental disorder defined by persistent and impairing difficulties in accurate or fluent word reading, poor decoding, and spelling, despite adequate intelligence, education, and intact sensory function (American Psychiatric Association, 2013). Impaired phonological awareness is regarded as the most predominant cause (Ramus, 2003). In China, DD affects around 3.9% to 9.7% of school-age children (Chan et al., 2007; Lin et al., 2020; Sun et al., 2013), significantly impacting their academic performance and future opportunities. Moreover, DD is often associated with other neurodevelopmental symptoms and generally extends to mental health issues (Sanfilippo et al., 2020), highlighting the important need to explore the broad effects of DD, particularly on mental health among school-age children. However, most dyslexia research has focused on Western, alphabetic-language contexts, with limited attention to Chinese settings. In China’s exam-driven, high-pressure educational environment, children with DD may be especially vulnerable (Xiao et al., 2022). Although inclusive education policies have been introduced in the broader special education field (Lei et al., 2025; Z. Wang et al., 2025), DD remains underrecognized in many regions. Understanding its intersection with mental health in this context is therefore critical for improving early identification and targeted support.

Mental health is a critical concern for school-age children, especially those with specific learning disorders like dyslexia (Georgiou et al., 2024). Dyslexia can lead to school failure and, subsequently, to emotional and behavioral problems. Depression, an essential determinant of mental health, is characterized by persistent sadness, emptiness, or irritability, accompanied by somatic and cognitive changes that severely impair daily functioning (American Psychiatric Association, 2013). Behavior problems, also closely linked to mental health, are typically classified into two dimensions: internalizing (anxiety, depression, somatic complaints, social withdrawal) and externalizing (delinquency, aggression) (Achenbach & Edelbrock, 1983). However, the relationship between reading ability and mental health problems has been debated (McArthur et al., 2022). What is not yet clear is the interconnections between dyslexia and its psychological implications in the Chinese context, particularly with regard to depressive symptoms and internalizing and externalizing behavior problems, as well as the existence of any potential mediating effects.

The association between cognitive abilities, such as reading ability, and increased degrees of negative mental health has garnered scholarly interest. Children with dyslexia exhibit a higher risk of comorbid depression (American Psychiatric Association, 2013), primarily due to frequent school failures that lower their self-esteem and increase frustration (Alexander-Passe, 2006), leading them to internalize such distress and pain. While research in alphabetic languages has shown the effects of poor reading on depressive symptoms (Vieira et al., 2024), there is much less information among Chinese children who engage with a logographic system. It has previously been observed that Chinese children with reading disabilities in grades 3 to 5 are more likely to be depressed than their non-disabled peers, influenced by the “unforgiving” Confucian culture that permeates the Chinese educational system (Li et al., 2024). Additionally, research from Taiwan noted that depression is more common in dyslexic children than it is in controls, with no differences found across sexes (L. C. Wang, 2021). Consequently, we can infer a significant negative association exists between reading ability and depressive symptoms among Chinese school-age children.

While the link between dyslexia and depressive symptoms has been explored, research has also provided important information on the co-occurrence of dyslexia and behavior problems, both internalizing and externalizing, adding to the psychological challenges faced by children with reading difficulties. For instance, a cross-sectional study from Norway discovered that children with dyslexia have higher rates of both internalizing and externalizing behavior problems than their peers, as assessed by the Child Behavior Checklist (CBCL) (Heiervang et al., 2001). Further investigation consistently suggests that dyslexic children experience more internalizing behavior problems (Kargiotidis & Manolitsis, 2024), with about half reporting bullying or teasing, which further weakens their self-concept and exacerbates these problems (Terras et al., 2009). It is worth mentioning that a recent meta-analysis by Vieira et al. (2024) identifies social withdrawal as the internalizing behavior problem generating the most profound effect in dyslexic children. Moreover, there seems to be an interconnection between internalizing and externalizing behavior problems, with dyslexic children exhibiting indications of both. Several researchers have confirmed the impact of reading disability on externalizing behavior problems, often related to comorbidity with Attention-Deficit/Hyperactivity Disorder (ADHD) (Rietz et al., 2012). In line with these findings, our published work has documented that Chinese school-age children with dyslexia achieve higher scores on conduct problems, learning problems, and hyperactivity compared to their typically developing peers (Y. Huang et al., 2020). However, despite these insights, the impact of reading ability on internalizing and externalizing behavior problems has yet to be closely examined in the Chinese educational setting. Hence, it could conceivably be hypothesized that reading ability correlates negatively with internalizing and externalizing behavior problems in Chinese school-age children.

The co-occurrence of depressive symptoms and behavior problems in typically developing children is well-documented and increases the chance of adverse outcomes (Caserini et al., 2023; Lau et al., 2023). Research demonstrates that behavior problems can lead to peer rejection and social isolation, resulting in depression (Gooren et al., 2011). A cohort study of Chinese school-age typically developing children found both externalizing and internalizing behavior problems as predictors of depressive symptoms (Leung et al., 2018), aligning with findings from the ALSPAC cohort which links early-onset conduct problems to later depression (Kretschmer et al., 2014). Notably, irritability, a component of externalizing behavior problems, is associated with increased depressive symptoms (Eisenberg et al., 2009), a trend also seen in children with ADHD who face higher rates of depressive symptoms (Joseph et al., 2019). Furthermore, genetics have been shown to account for the progression from early externalizing to later internalizing disorders like depression, according to Wertz et al. (2015). Based on these findings, one hypothesis to be tested is that internalizing and externalizing behavior problems significantly contribute to the development of depressive symptoms in Chinese school-age children. At the same time, previous studies have revealed diverse trajectories between depressive symptoms and behavior problems among school-age children. Historically known as “masked depression”, children with depression frequently display comorbid behavior problems like aggression, hyperactivity, and somatic complaints, which differ from typical adult depressive symptoms (Cytryn & McKNEW, 1972). Additionally, irritability induced by depression can intensify these behavior problems (Wolff & Ollendick, 2006). Longitudinal research (e.g., Ritakallio et al., 2008) has shown that increased depressive symptoms can predict future behavior problems, hinting at a reciprocal relationship. Therefore, another hypothesis to be examined is that depressive symptoms positively influence internalizing and externalizing behavior problems, particularly in Chinese children with dyslexia. Despite the literature surrounding the mutual influence of depressive symptoms and behavior problems (Caserini et al., 2023; Lau et al., 2023), there remains a lack of evidence specifically on their bidirectional effects in dyslexic children. Hence, this research assumes the potential bidirectional relationship between depressive symptoms and internalizing/externalizing behavior problems in Chinese school-age children with dyslexia.

In mediation theory, a mediator explicates how a predictor influences an outcome, transforming statistical associations into theoretically grounded mechanisms (Baron & Kenny, 1986). From a developmental-transactional perspective, low academic competence can erode self-worth and trigger maladaptive coping. Accordingly, depressive symptoms and behavior problems may not simply co-occur with reading difficulties; they may function as pathways through which academic struggles affect broader adjustment. Framing these psychological constructs as mediators reveals critical leverage points for early intervention. When attempting to clarify the mechanisms underlying academic functioning and externalizing or internalizing behavioral symptoms, Moilanen et al. (2010) proposed the academic incompetence hypothesis: students with low academic competence may engage in abnormal behavior problems to express distress and defend their self-worth. Inspired by this, we consider behavior problems as a mediator between reading ability and depressive symptoms, hypothesizing that dyslexia, marked by limited reading ability, could lead to internalizing and externalizing behavior problems and, in turn, depressive symptoms. Although direct evidence is lacking, indirect support from Xiao et al. (2022) demonstrates that in Chinese children, the potential impact of dyslexia on depressive symptoms may be mediated by time spent on homework and stress. Thus, the key research question of this study is whether internalizing and externalizing behavior problems mediate the effect of reading ability on depressive symptoms.

Conversely, previous research has found that about half of the children with specific learning disorders show depression at initial evaluation, suggesting that behavior problems might stem from either learning disabilities or depressive moods (Weinberg et al., 1989). In light of this, we anticipate that depressive symptoms might partially mediate the association between reading ability and behavior problems, implying that poorer reading skills could lead to more severe depressive symptoms and, subsequently, more serious behavior problems. However, up to the present day there has been little discussion about the potential mediation effect of reading ability on behavior problems, both internalizing and externalizing. Thus, another significant research question of this study is whether depressive symptoms mediate the impact of reading ability on internalizing and externalizing behavior problems.

Existing research recognizes that reading difficulties can lead to emotional and behavioral disturbances, which may, in turn, hinder one’s capacity to engage with and succeed in reading tasks, creating a cycle of mutual influence (Burke et al., 2015). Depressive symptoms can significantly reduce motivation, concentration, and memory (Ingoldsby et al., 2006), essential for effective reading, whereas behavior problems may interfere with the reading process due to poor attention, impulsivity, and social challenges within educational settings (Spira & Fischel, 2005). Conversely, difficulties in reading challenges can heighten frustration, lower self-esteem, and increase academic disengagement, further worsening both depressive symptoms and behavior problems. This cycle of mutual influence underscores the importance of early detection and intervention. Longitudinal evidence, however, suggests the relationship is not fully reciprocal: reading deficits more consistently predict later behavior problems than the reverse, and only specific behavioral domains such as poor task engagement, rather than broader internalizing or externalizing problems, appear to impede subsequent reading growth (Morgan et al., 2008). Therefore, this study aims to address the following research question: *Do depressive symptoms and internalizing and externalizing behavior problems interact with reading ability among Chinese school-age children?*

**Figure 1:**
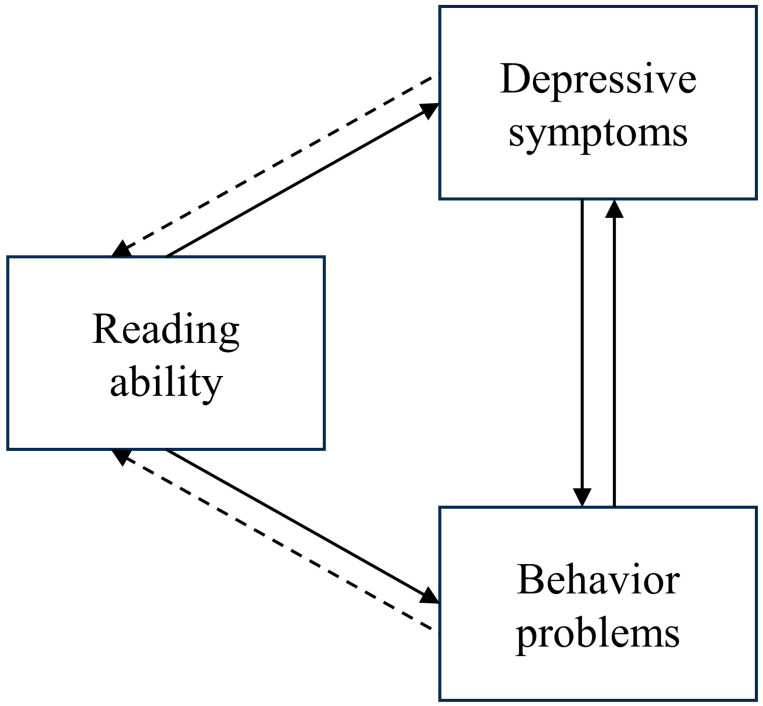
Hypothesized model of mediating loop. Notes: Behavior problems represents two different variables: internalizing behavior problems and externalizing behavior problems.

In summary, there are two primary aims of this study: 1. to investigate the presence of depressive symptoms and internalizing and externalizing behavior problems among Chinese school-age children with dyslexia, and 2. to ascertain the potential pathways linking reading ability with depressive symptoms and internalizing and externalizing behavior problems. Illustrated in Figure 1 is a hypothesized model of the mediating loop that conceptualizes the anticipated relationships. In this study, the impact of dyslexia on mental health will be explored, mainly focusing on the mediating roles of depressive symptoms and internalizing and externalizing behavior problems. Furthermore, this study sets out to explore the empirical evidence of the bidirectional relationship between reading ability and mental health, including depressive symptoms and internalizing and externalizing behavior problems, highlighting the importance of early detection and intervention. Specifically, the following hypotheses will be examined among Chinese school-age children:

**H1.**  *Reading ability negatively correlates with depressive symptoms (i.e., lower reading ability is linked to higher levels of depressive symptoms).*

**H2.**  *Reading ability negatively correlates with internalizing/externalizing behavior problems (i.e., lower reading ability is linked to more severe behavior problems).*

**H3.**  *Depressive symptoms and internalizing/externalizing behavior problems mutually influence each other (i.e., higher levels of depressive symptoms are related to more severe internalizing/externalizing problems, and vice versa).*

**H4.**  *Internalizing/externalizing behavior problems mediate the relationship between reading ability and depressive symptoms (i.e., lower reading ability is associated with more severe behavior problems, which in turn are associated with higher depressive symptoms).*

**H5.**  *Depressive symptoms mediate the relationship between reading ability and internalizing/externalizing behavior problems (i.e., lower reading ability is associated with higher depressive symptoms, which in turn are associated with more severe behavior problems).*

**H6.**  *Reading ability and mental health symptoms (depressive symptoms and internalizing/externalizing behavior problems) are mutually associated (i.e., lower reading ability is linked to worse mental health, and vice versa).*

## 2. Materials and Methods

### 2.1. Participants

The study adopts a case–control approach. The case group consists of 44 children diagnosed with dyslexia at the Children and Adolescents Outpatient Clinic of Shantou University Mental Health Center from December 2021 to December 2023. Diagnoses were confirmed by two senior child psychiatrists using the Chinese Reading Ability Test (CRAT) (A. Huang et al., 2020) and Diagnostic and Statistical Manual of Mental Disorders, Fifth Edition (DSM-5) (American Psychiatric Association, 2013) criteria. Specifically, a diagnosis of dyslexia required a CRAT standard score of at least 1 standard deviation below the age-appropriate mean, as well as confirmation of DSM-5 criteria for Specific Learning Disorder with impairment in reading. When the diagnosis was uncertain, a third psychiatrist was consulted to confirm it. Criteria for selecting the participants were as follows: (1) attendance in grades 2 through 5, (2) aged 7 to 12, (3) an IQ above 85, as determined by the Raven’s Standard Progressive Matrices, and (4) a Chinese language exam score in the bottom 20% of their class. Participants were excluded if they had neurological disorders (e.g., epilepsy), suspected brain injury, uncorrected sensory impairment (vision or hearing), or intellectual disability. After that, a random sample of 81 students from a public primary school in Shantou was recruited as a control group. Each dyslexic child was matched with up to two controls of the same grade and within ± 6 months of age. All controls did not meet the diagnostic or inclusion criteria for dyslexia. In total, the study included 125 participants: 44 children with dyslexia and 81 typically developing children.

The sample size was calculated using G*Power 3.1.9.7 (Faul et al., 2009). Default parameters were applied: *f*^2^ = 0.15 (medium effect size), α = 0.05, and power (1 − β) = 0.95. The number of predictors was set at 2, depending on the hypothesized model. A priori power analysis indicated a minimum sample size of 107 to achieve sufficient statistical power. Subsequently, a post hoc power analysis conducted with the actual sample size of 125 yielded a power of 0.98, well above the recommended minimum value of 0.80. Consequently, 125 children were successfully recruited, ensuring robust statistical validity.

### 2.2. Measures

#### 2.2.1. Reading Ability

Phonological processing, the core deficit in dyslexia, is crucial for reading (Siegel, 1993). Studies in alphabetic languages have confirmed the phonological deficit theory of dyslexia, showing its predictive value for reading ability (Christoforou et al., 2023). Despite differences in logographic languages like Chinese, a growing body of research has identified a strong correlation between phonological awareness and reading ability (J. Wang et al., 2021), further supported by neuroimaging evidence of phonological processing deficits in Chinese dyslexia (Cao et al., 2017). Therefore, we assessed children’s reading performance by measuring their phonological processing skills.

**Figure 2:**
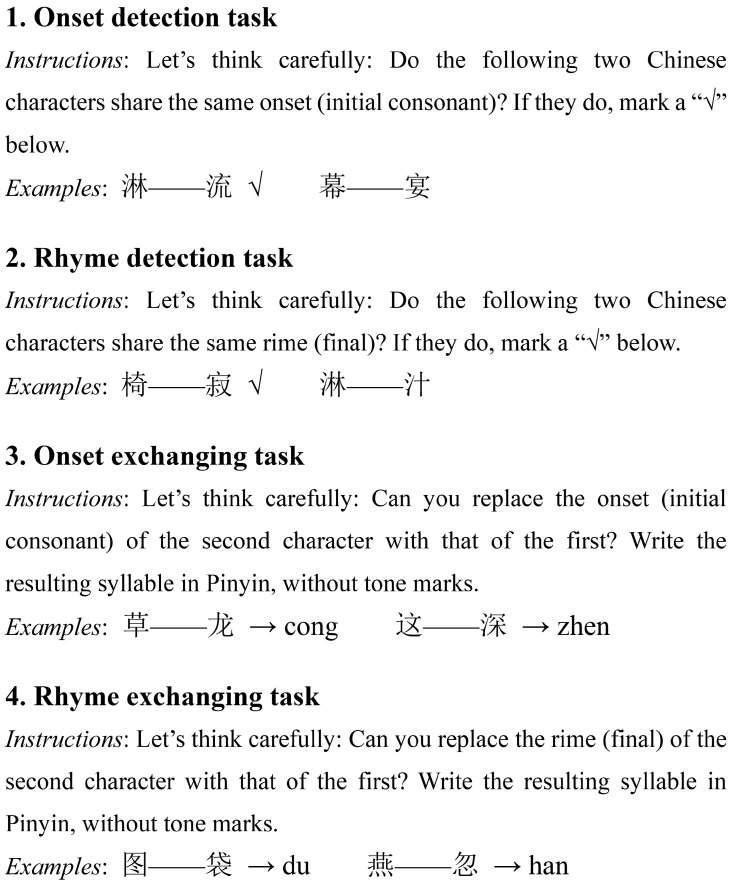
Sample items from four phonological processing tasks. Notes: All items were administered in Mandarin Chinese. English instructions were translated from the original Chinese used during testing.

To investigate phonological awareness among Chinese school-age children, we modified phonological processing tasks developed by Song (2006) according to the unique characteristics of Chinese characters. These tasks encompass phonological recognition, including onset and rhyme detection, and phonological exchanging, involving onset and rhyme exchanging. In the phonological recognition task, participants were tasked with identifying whether pairs of Chinese characters possessed matching initials or rhymes. The stimuli, consisting of eighty characters selected from a second-grade Chinese language textbook published by People’s Education Press, were curated into twenty sets designed to reflect similarities in structural composition and stroke patterns, facilitating the execution of both phonological tasks. Each correct response was scored as 1 point, and incorrect responses as 0, resulting in a total score for onset detection and rhyme detection. For the phonological exchanging task, using the same textual material and selection criteria, children were required to exchange consonants and vowels between pairs to form new characters. For instance, in the onset exchanging task, the onset (initial consonant) of one character was swapped with the onset of another, while in the rhyme exchanging task, the rhyme (final vowel) was swapped. Each correct exchange was worth 1 point, with a maximum score of 20 points for both onset and rhyme exchanging tasks. Experts thoroughly explained the tasks to the children and conducted practice sessions to ensure complete comprehension. Illustrative examples of all four phonological processing tasks are presented in Figure 2. Reading ability was assessed by the percentage of correct responses for each test, including onset detection, rhyme detection, onset exchanging, and rhyme exchanging. A composite score for the phonological processing tasks was calculated based on the percentage of correct responses across all tasks, reflecting the overall accuracy of the children’s reading performance. The reliability of these tasks in our sample was confirmed with a Cronbach’s α of 0.941.

#### 2.2.2. Depressive Symptoms

The Depression Self-Rating Scale for Children (DSRS) (Birleson, 1981) was employed to assess children’s self-reported depressive symptoms. It has eighteen items scored on a 0–2 Likert scale, 0 for “never,” 1 for “sometimes,” and 2 for “most of the time.” Scores of 15 or above suggest possible depression, with higher scores being more severe. The DSRS is widely used in China, showing good reliability (Cronbach’s α = 0.871) in this study.

#### 2.2.3. Behavior Problems

The Child Behavior Checklist for ages 6–18 (CBCL/6–18) (Achenbach & Edelbrock, 1983), filled out by parents, is a standardized tool for assessing behavior problems in school-age children and is appropriate for Chinese children with excellent reliability and validity. It consists of 113 issue items covering various behavior problems, divided into internalizing (anxious/depressed, somatic complaints, withdrawn), and externalizing (delinquent, aggressive) scales. A three-step response scale (0, 1, 2) is chosen, with 0 representing “not true,” 1 representing “somewhat or sometimes true,” and 2 representing “very true or often true.” Increased scores indicate more severe behavior problems. In this study, each scale demonstrated strong reliability, with a Cronbach’s α of 0.834 for the internalizing scale and 0.868 for the externalizing scale. 

### 2.3. Statistical Analyses

Descriptive statistics were calculated: mean and standard deviation (Mean ± SD) for normally distributed variables; median and interquartile ranges (M, P_25_, P_75_) for non-normally distributed variables; and frequency and percentage (*n*, %) for categorical variables. The dyslexic and control groups were compared using the independent samples *t*-test, the Mann–Whitney *U* test, and the Chi-square test.

Prior to analyses, we examined key statistical assumptions. Normality was assessed using the Kolmogorov–Smirnov test. Reading ability was approximately normally distributed (*p* > 0.05), while depressive symptoms, internalizing, and externalizing behavior scores significantly deviated from normality (*p* < 0.05). Consequently, Spearman correlation analysis was chosen to examine the strength of relationships between reading ability, depressive symptoms, and internalizing and externalizing behavior problems. Multicollinearity was ruled out by confirming that all Variance Inflation Factors (VIFs) were below 5. Moreover, stratified analysis by sex was performed to explore the impact of sex on mental health indicators.

Additionally, Exploratory Graph Analysis (EGA) was employed to identify distinct dimensions within the DSRS, enabling a more nuanced comparison of mood differences between children with and without dyslexia. We conducted EGA using the *EGAnet* R package within R version 4.4.1, applying the graphical lasso (glasso) model combined with the walktrap community detection algorithm to cluster items (Golino et al., 2020). To verify the stability of the identified structure, bootstrap EGA (bootEGA) was performed with 5000 resamples. Stability was assessed through key metrics: the frequency of consistently detecting the same number of dimensions, the item replication index (frequency of consistent item assignment to their respective dimensions), and structural consistency within dimensions, with all metrics meeting an acceptability threshold of >0.75 (Wu et al., 2024).

In order to examine the mediating effect, we used multiple linear regressions through model 4 of the PROCESS macro version 4.1 for SPSS (Hayes, 2017). Model 4 was selected because it is specifically designed to estimate the indirect effect of a single mediator between an independent and a dependent variable, aligning with our study’s focus on examining specific mediating pathways. As one of the most effective methods for testing mediation, the bias-corrected percentile bootstrap method is a non-parametric approach that allows for exploring indirect effects, even with limited sample sizes (Preacher & Hayes, 2008). This analysis was conducted with 5000 bootstrap samples to ensure robustness. A statistically significant indirect effect was determined if the 95% confidence interval (CI) did not include 0.

We pre-specified eight single mediator models (Models A–H). Each model examines a bidirectional pathway between reading ability and mental health indicators. Models A–D treat reading ability as the predictor (X) and test depressive symptoms or internalizing/externalizing behavior problems as mediators (M); models E–H reverse the direction, treating each mental health indicator as X and reading ability as Y. Table 1 summarizes the independent variable, mediator and dependent variable for every model.

All analyses were carried out using IBM SPSS Statistics version 26.0 and R version 4.4.1. A *p* value < 0.05 was considered significant.

## 3. Results

### 3.1. Demographic Characteristics of the Participants

Table 2 shows demographic characteristics for 44 dyslexic children and 81 typically developing children across grades 2 to 5. The average age of the dyslexic group was 9.57 ± 1.21 years, compared to 9.20 ± 1.24 years for the control group. Dyslexic children had a higher proportion of boys and significantly lower parental education and family income levels than controls (all *p* < 0.05).

### 3.2. Reading Ability: Dyslexic and Control Group Comparisons

**Figure 3:**
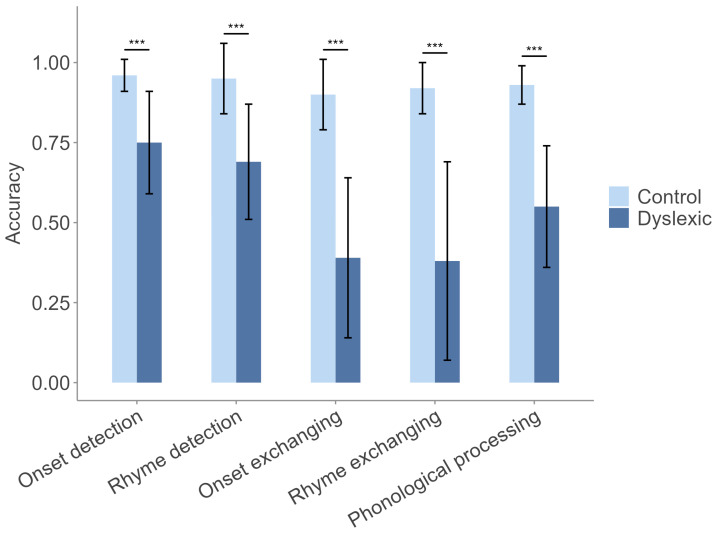
Comparison of reading ability between the dyslexic and control groups. Notes: *** *p* < 0.001.

As shown in Figure 3, our assessment found significant differences in reading ability between the dyslexic and control groups across all tasks (all *p* < 0.001). Children with dyslexia performed much worse in onset and rhyme detection, with even larger gaps in more complex tasks like onset and rhyme exchanging. A comprehensive examination of phonological processing further confirmed substantial deficits in dyslexic children, suggesting clear disparities in reading ability.

### 3.3. Depressive Symptoms: Exploratory Graph Analysis and Group Comparisons

**Figure 4:**
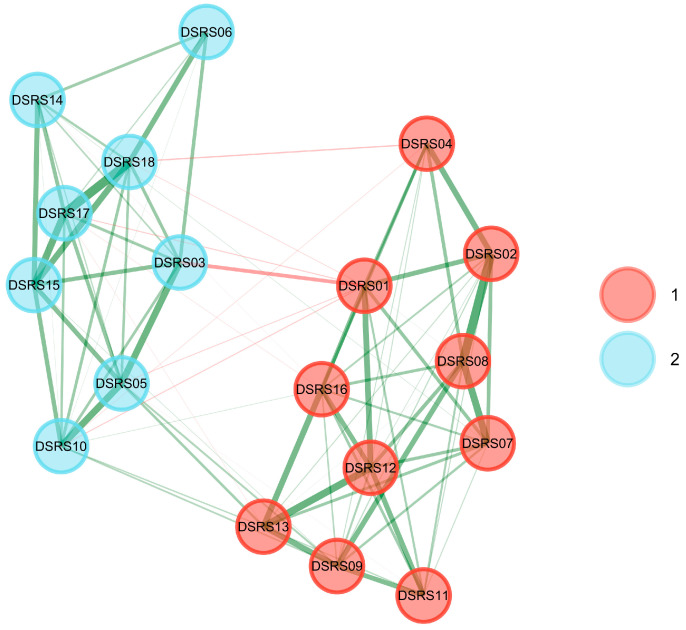
DSRS network displaying the EGA-identified dimensional factors. Notes: EGA = Exploratory Graph Analysis. Green edges indicate positive associations, red edges indicate negative ones, and edge thickness reflects the strength of the connection. *Low Positive Affect*: DSRS01 = I look forward to things as much as I used to; DSRS02 = I sleep very well; DSRS04 = I like to go out and play; DSRS07 = I have lots of energy; DSRS08 = I enjoy my food; DSRS09 = I can stick up for myself; DSRS11 = I am good at things I do; DSRS12 = I enjoy the things I do as much as I used to; DSRS13 = I like talking with my family; DSRS16 = I am easily cheered up. *Negative Affect*: DSRS03 = I feel like crying; DSRS05 = I feel like running away; DSRS06 = I get tummy aches; DSRS10 = I think life is not worth living; DSRS14 = I have horrible dreams; DSRS15 = I feel very lonely; DSRS17 = I feel so sad that I can hardly stand it; DSRS18 = I feel very bored.

**Figure 5:**
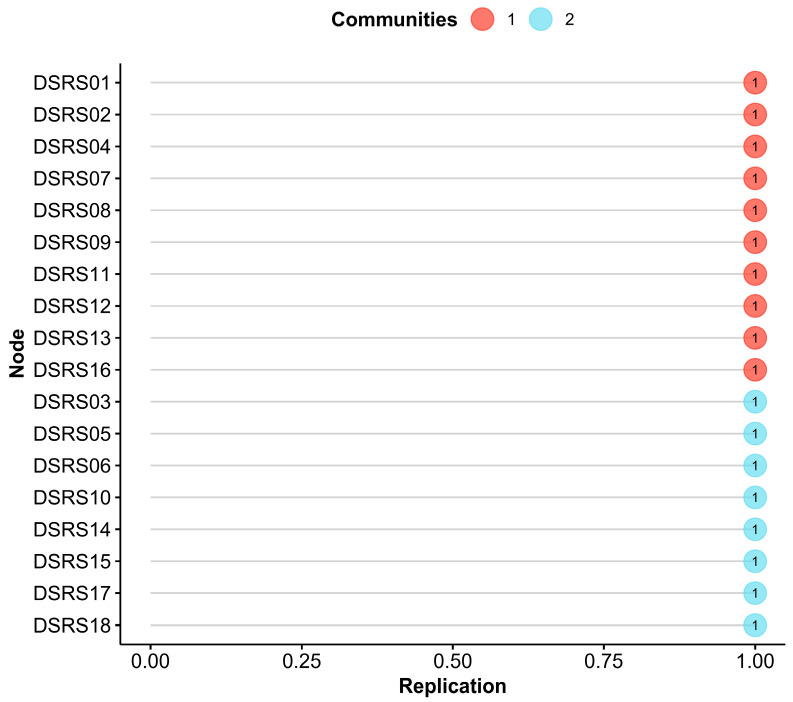
EGA network item stability. Notes: The average item stability of cluster one and two was 1.000 and 1.000.

To explore the underlying structure of depressive symptoms in Chinese school-age children, Exploratory Graph Analysis (EGA) was applied to the DSRS. EGA identified two dimensions (Figure 4): *Low Positive Affect* (items DSRS01, DSRS02, DSRS04, DSRS07, DSRS08, DSRS09, DSRS11, DSRS12, DSRS13, and DSRS16) and *Negative Affect* (items DSRS03, DSRS05, DSRS06, DSRS10, DSRS14, DSRS15, DSRS17, and DSRS18). In the network visualization, nodes represent the 18 items of the scale, green edges denote positive associations, red edges represent negative associations, and edge thickness reflects association strength. Bootstrap analyses of EGA revealed highly stable results, with both dimensions consistently detected at a frequency of 1.000. Each item was exclusively assigned to its respective dimension with a frequency of 1.000 (Figure 5), and structural consistency was perfect (1.000), indicating excellent performance.

As shown in Table 3, children with dyslexia had a higher prevalence of possible depression (40.9% vs. 17.3%, *p* < 0.01) and significantly elevated DSRS scores (*p* < 0.001). They also reported higher levels of both *Low Positive Affect* and *Negative Affect* (*p* < 0.001 for both), indicating greater emotional difficulties.

### 3.4. Behavior Problems: Dyslexic and Control Group Comparisons

The Kolmogorov–Smirnov test indicated non-normal distributions for CBCL scores (*Z*_Internalizing_ = 0.260, *Z*_Externalizing_ = 0.277, both *p* < 0.001), leading to the use of the Mann–Whitney *U* test for comparisons. As detailed in Table 4, significant differences were found in internalizing and externalizing behavior problems (all *p* < 0.001), showing increased behavioral challenges among dyslexic children.

### 3.5. Spearman Correlation Analysis: Reading Ability and Mental Health Indicators

Spearman’s rank-order correlation was selected due to the non-normal distribution of depressive symptoms and internalizing and externalizing behavior problems, making it a more appropriate approach than the Pearson correlation. Table 5 presents the results of Spearman’s Rho analysis, which showed significant inverse relationships between reading ability and depressive symptoms, as well as internalizing and externalizing behavior problems, while positive correlations between depressive symptoms and internalizing and externalizing behavior problems (all *p* < 0.01). Stratified by sex, significant correlations were found across all variables for boys (all *p* < 0.01; see Table S1), while for girls, reading ability only significantly negatively correlated with internalizing and externalizing behavior problems (both *p* < 0.01) but not depressive symptoms (*p* > 0.05; see Table S2).

### 3.6. Mediation Analyses of Reading Ability and Mental Health

**Figure 6:**
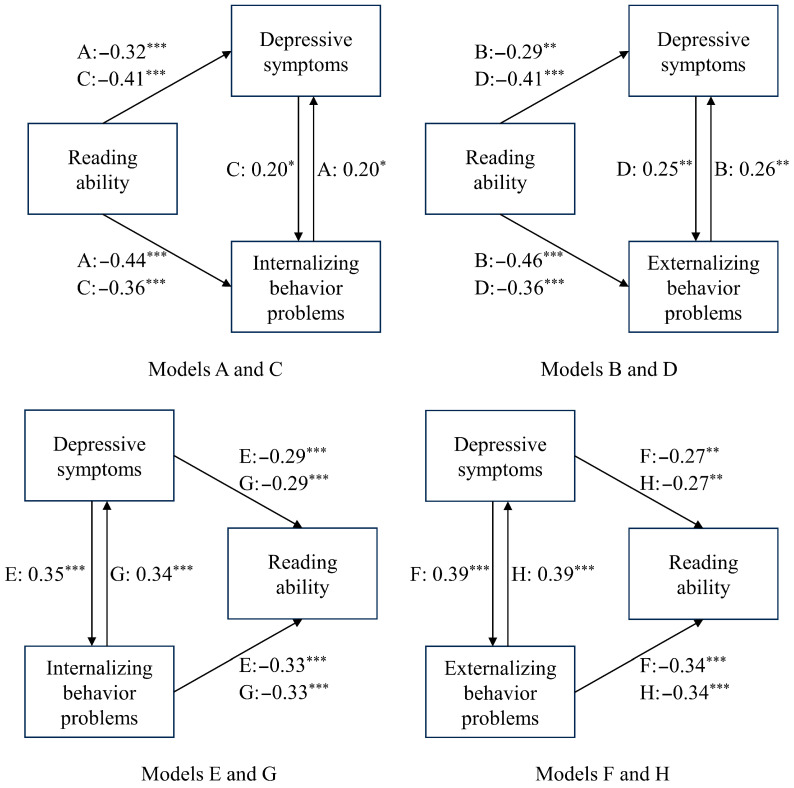
Mediation analyses of reading ability and mental health. Notes: The regression coefficients were given to the antecedent variables in *Models A–H*. *Models A and C*: Depressive symptoms and internalizing behavior problems served as mutual mediators of the effects of reading ability on each other; *models B and D*: depressive symptoms and externalizing behavior problems served as mutual mediators of the effects of reading ability on each other; *models E and G*: depressive symptoms and internalizing behavior problems served as mutual mediators of each other’s effects on reading ability; *models F and H*: depressive symptoms and externalizing behavior problems served as mutual mediators of each other’s effects on reading ability. * *p* < 0.05, ** *p* < 0.01, *** *p* < 0.001.

As shown in Figure 6 and Table 6, eight mediation models were used to explore the potential pathways relating reading ability with depressive symptoms and internalizing and externalizing behavior problems. Sex and age were adjusted as covariates, and all variables were centralized and standardized using *Z*-scores.

#### 3.6.1. Influence of Reading Ability on Depressive Symptoms and Behavior Problems

(1) Models A-B: behavior problems as mediators

As can be seen from Table 7, adjusted for sex and age, the direct effect of reading ability on depressive symptoms was significant (β = −0.41, *p* < 0.001). Reading ability was also found to negatively predict internalizing (β = −0.44), and externalizing behavior problems (β = −0.46) (both *p* < 0.001), which were positively associated with depressive symptoms (β = 0.20, 0.26, respectively, both *p* < 0.05). Moreover, mediation analysis claimed behavior problems as key mediators, with the indirect impact of internalizing (indirect effect = −0.090, 95%CI: −0.171, −0.026) and externalizing behavior problems (indirect effect = −0.119, 95%CI: −0.231, −0.044) explaining 22.03% and 29.12% of the total effect. Interestingly, externalizing behavior problems provided the strongest mediation effect.

(2) Models C-D: depressive symptoms as mediators

Table 8 illustrates that reading ability had a significant direct impact on behavior problems, and negatively predicted depressive symptoms, which in turn positively related to behavior problems (β = 0.20 for internalizing, and β = 0.25 for externalizing, both *p* < 0.05). Strong evidence was found that depressive symptoms partially mediated the effect of reading ability on behavior problems. Specifically, the indirect effects through internalizing (indirect effect = −0.083, 95%CI: −0.165, −0.022) and externalizing behavior problems (indirect effect = −0.102, 95%CI: −0.187, −0.035) accounted for 18.78%, and 22.23% of the total effect, with the latter showing the most noticeable mediation by depressive symptoms.

#### 3.6.2. Influence of Depressive Symptoms and Behavior Problems on Reading Ability

(1) Models E-F: behavior problems as mediators

It is apparent from Table 9 that depressive symptoms directly predicted reading ability (β = −0.40, *p* < 0.001), and significantly influenced behavior problems: internalizing (β = 0.35) and externalizing (β = 0.39) (both *p* < 0.001), which were negatively correlated with reading ability (β = −0.33, −0.34, both *p* < 0.001). Additionally, the effect of depressive symptoms on reading ability was partially mediated by behavior problems. The indirect effects were −0.114 for internalizing (95%CI: −0.193, −0.054) and −0.136 for externalizing behavior problems (95%CI: −0.233, −0.066), accounting for 28.24% and 33.53% of the total effect, with externalizing exhibiting the highest mediation effect.

(2) Models G-H: depressive symptoms as mediators

What stands out in Table 10 demonstrates the direct impact of behavior problems on reading ability (β = −0.43 for internalizing and β = −0.45 for externalizing, both *p* < 0.001). Also, behavior problems’ positive relation to depressive symptoms (β = 0.34, 0.39, both *p* < 0.001) translated into a negative association with reading ability (β = −0.29, −0.27, both *p* < 0.01). Depressive symptoms acted as a mediator, with indirect effects of internalizing (indirect effect = −0.099, 95%CI: −0.190, −0.036) and externalizing behavior problems (indirect effect = −0.105, 95%CI: −0.202, −0.030), with each explaining around 23% of the total effect, illustrating a consistent mediation effect across internalizing and externalizing behavior problems.

## 4. Discussion

In the current study, we set out to explore the interconnections between dyslexia and its psychological implications among Chinese school-age children, specifically focusing on depressive symptoms and internalizing and externalizing behavior problems. By comparing children with dyslexia to those without, we examined the prevalence of these psychological issues. Through mediation analysis, we investigated the interrelations hypothesizing that dyslexia impacts mental health via the mediating effects of depressive symptoms and internalizing and externalizing behavior problems. Furthermore, our research sought to illuminate the bidirectional correlation between reading ability and mental health, making an original contribution to the potential mediating loop.

The first part of our investigation reaffirmed that children with dyslexia perform more poorly on phonological processing tasks and that the gap widens as linguistic demands increase, supporting the phonological deficit account of dyslexia (Christoforou et al., 2023; J. Wang et al., 2021; Song, 2006). Moreover, academic struggles associated with dyslexia are not isolated but are closely linked with higher incidences of depressive symptoms and behavior problems, consistent with recent studies indicating the implications of dyslexia extend beyond academic performance to affect mental health (Georgiou et al., 2024). This finding is consistent with that of De Lima et al. (2020), who reported that parents of children with dyslexia observe significantly more behavior problems and that these children exhibit higher levels of depressive symptoms. Notably, De Lima et al. (2020) also revealed a discrepancy between children’s self-reported depressive symptoms and parent-reported internalizing behaviors, suggesting potential differences in perceived emotional challenges. To further explore the diverse characteristics of depressive symptoms, we performed Exploratory Graph Analysis, which supported a two-factor model comprising *Low Positive Affect* and *Negative Affect* (Green et al., 2024). Dyslexic children showed elevated scores on both dimensions, suggesting that emotional blunting and negative mood may represent distinct yet co-occurring pathways of vulnerability. Additionally, the pattern of behavior problems observed is consistent with previous reports (Sharma et al., 2018), reinforcing the need for interventions that address both learning difficulties and co-occurring psychological challenges. These findings draw our attention to the multifaceted burden of dyslexia and emphasize the importance of comprehensive screening and support strategies that extend beyond literacy skills alone.

Another finding is that all variables—reading ability, depressive symptoms, internalizing, and externalizing behavior problems—were strongly interrelated, with substantial sex differences: in boys, reading ability correlated with all variables, while in girls, it was linked only to internalizing and externalizing behavior problems, not depressive symptoms. These sex differences are difficult to explain but may result from girls internalizing their struggles or being less likely to report depressive symptoms tied to academics, with their depressive symptoms potentially more influenced by psychosocial factors like stress and social support (Schraedley et al., 1999). A similar pattern of sex differences was observed by Xiao et al. (2022), where dyslexia in boys was correlated with all psychological outcomes, whereas in girls, dyslexia showed no significant connections with either anxiety or stress symptoms. In contrast, Kiuru et al. (2011) found a more vital link between learning challenges and depressive symptoms in Finnish girls aged 15–16 than in boys, suggesting that cultural expectations and developmental stages may play a role. These findings emphasize the need for sex-sensitive interventions that account for the different ways boys and girls respond to reading difficulties and their psychological implications, while also calling for further research into the underlying mechanisms driving these sex-based differences.

As anticipated, this study confirmed the direct relationship between reading ability and depressive symptoms, in agreement with a follow-up study in China (Xiao et al., 2023). Children with dyslexia often experience chronic stress and frustration, leading to negative self-perception and reduced self-esteem, which significantly elevate their risk for depression. Neurobiological studies suggest that dyslexia is associated with altered activity in the prefrontal cortex and limbic system (Al Dahhan et al., 2020), promoting emotional dysregulation and heightened responses to stress and failure that may foster depressive symptoms (Pizzagalli & Roberts, 2022). Moreover, chronic stress among dyslexic children might result in the dysregulation of the hypothalamic–pituitary–adrenal (HPA) axis (Zakopoulou et al., 2019), further increasing susceptibility to depression (DeMorrow, 2018). Importantly, the cultural dimensions of these findings deserve specific consideration. The Confucian culture prevalent in Chinese educational contexts emphasizes academic achievement and diligence, which may intensify feelings of inadequacy and self-blame among children facing reading challenges. Unlike many Western contexts, where individual differences and diverse talents may receive more recognition, Confucian-influenced systems tend to define success through scholastic performance, exacerbating internalized distress for those who cannot meet such expectations (Li et al., 2024). In addition, collectivist values inherent in Confucian culture heighten sensitivity to social comparisons and perceived failures, potentially magnifying internalizing symptoms among dyslexic children who struggle to conform academically. Likewise, this study established direct correlations between reading ability and internalizing and externalizing behavior problems, in line with observations that comorbid behavior problems are prevalent among dyslexic children (Eman, 2024). In cultures that highly value academic achievement, children who struggle with reading may be more susceptible to bullying or social isolation, exacerbating these behavior problems (Terras et al., 2009). Additionally, reading disabilities may reflect broader weaknesses in executive functions (Basharpoor et al., 2024), such as inhibitory control, working memory, and cognitive flexibility, which can also manifest as behavior problems (Likhitweerawong et al., 2024). Thus, cultural expectations not only intensify internal distress but may also contribute to external behavioral responses. Future research should continue exploring these cultural nuances by comparatively analyzing mental health problems across different cultural and linguistic contexts to better tailor culturally sensitive interventions.

Consequently, significant correlations have been established between reading ability and both depressive symptoms and behavior problems; however, these relationships have seldom been examined concurrently. Moreover, the potential mediating mechanisms underlying these correlations remain unexplored. To address this, mediation analyses were conducted to determine whether depressive symptoms and behavior problems mutually mediate the relationship between dyslexia and mental health issues. One interesting finding is that both internalizing and externalizing behavior problems mediated the impact of reading ability on depressive symptoms. Notably, externalizing behavior problems emerged as the strongest mediator, explaining 29.12% of the total effect. This may be linked to the comorbidity of dyslexia and ADHD, which often presents as impulsivity and poor emotional self-regulation—traits that disrupt classroom learning and peer relationships, leading to greater frustration and social exclusion, both known risk factors for depression. This aligns with research by Kjeldsen et al. (2016), which suggests that externalizing problems could disrupt social interactions and reduce well-being, considerably exacerbating depressive symptoms. Furthermore, because externalizing behaviors are more visible and disruptive in school settings, they are more likely to elicit negative feedback loops involving academic failure, punitive responses, and emotional distress, which may explain their stronger mediating role. Another important finding is that depressive symptoms themselves also mediated the relationship between reading ability and internalizing and externalizing behavior problems, most significantly affecting externalizing behaviors, which account for 22.23% of the effect. These observations suggest a bidirectional relationship, where not only do behavior problems mediate the effects of reading ability on depressive symptoms, but depressive symptoms also influence the manifestation of behavior problems, potentially through mechanisms of emotional dysregulation (Bunford et al., 2015). Previous research has noted reading ability as a protective factor against mental health challenges (Hyseni Duraku et al., 2023). Our study builds on this by quantifying the mediation effects of internalizing and externalizing behavior problems and depressive symptoms, highlighting the potential benefits of promoting reading skills as preventive measures. Furthermore, interventions for behavior problems may benefit from incorporating strategies to improve reading ability, potentially reducing their impact on depression and vice versa.

In addition to clarifying the mediating roles of behavior problems and depressive symptoms between reading ability and mental health, the results also indicated how mental health issues inversely influence reading ability through these same mediators. Depressive symptoms directly reduced reading ability while also exacerbating internalizing and externalizing behavior problems, which further impaired reading skills. Moreover, models examining depressive symptoms as mediators showed that internalizing and externalizing behavior problems not only directly decreased reading ability but also contributed to heightened depressive symptoms, which in turn, worsened reading outcomes. According to these data, we can infer that depressive symptoms may impair critical cognitive functions for reading, such as concentration, memory, and information processing speed, corroborating findings by Hack et al. (2023). Additionally, behavior problems can disrupt classroom learning and peer interactions, essential for reading development, as demonstrated by Morgan et al. (2008). Therefore, interventions that address both emotional well-being and behavior management are likely to enhance reading achievements significantly.

Our study reveals a cyclical and mutually reinforcing relationship between reading ability, depressive symptoms, and internalizing and externalizing behavior problems among Chinese school-age children, which together perpetuate both academic and psychological challenges. It can be suggested that depressive symptoms and behavior problems both stem from and contribute to reading challenges, creating a feedback loop that intensifies without intervention. Addressing any single aspect of this triad—reading ability, depressive symptoms, or behavior problems—in isolation may not be sufficient. Instead, interventions that target all three areas could be more effective. This highlights the importance of adopting multidisciplinary approaches that integrate educational and psychological expertise to design more comprehensive and sustainable support strategies for children (Vostrý et al., 2022). Personalized intervention plans should be tailored to the unique dyslexia phenotypes, emotional states, and behavioral characteristics of each child. For example, evidence-based reading programs such as phonological training or fluency training can directly address reading difficulties (Ruan et al., 2024), while psychological therapies like cognitive behavioral therapy (CBT) and mindfulness meditation can help manage depressive symptoms and behavior problems (Hendren et al., 2018). In particular, managing externalizing behavior problems in dyslexic children is crucial for tackling the reading and emotional challenges they face due to the comorbidity of dyslexia and ADHD. Moreover, innovative technologies like transcranial magnetic stimulation (TMS) (Arrington et al., 2023), virtual and augmented reality (VR/AR) (Hussain et al., 2023; Maresca et al., 2024), and artificial intelligence (AI) tools (Goodman et al., 2024), could be used to personalize the learning experience and boost engagement. School-based social–emotional learning (SEL) programs can further foster emotional resilience and help children better cope with academic and social pressures (Corcoran et al., 2018). By integrating reading support with psychological assistance, such holistic interventions could create an environment where children can thrive academically and psychologically, laying the foundation for robust educational outcomes and social development.

Several limitations to this study need to be acknowledged. First, the case–control design limits the potential to draw inferences regarding causality among reading ability, depressive symptoms, and behavior problems, suggesting the necessity for longitudinal research to explore the temporal sequence and causal pathways more thoroughly. Specifically, future investigations should focus on the longitudinal tracking of dyslexia, its evolution over time, and its psychological correlates. It is also essential to scrutinize the extent to which mental health issues may contribute to or exacerbate reading difficulties. Such research will be pivotal for elucidating the directional relationships between these variables and pinpointing optimal windows for intervention strategies that could yield the most significant improvements in outcomes. Second, although the study is adequately powered, the limited sample size and the exclusive focus on Chinese school-age children may restrict the generalizability of the findings to broader populations. Research on the cultural diversity of dyslexia has shown that, despite dyslexics across different writing systems sharing a common neural basis (Hu et al., 2010), differences in orthographies contribute to disparities in reading performance (Paulesu et al., 2001). On the other hand, the emotional and behavioral challenges associated with dyslexia may manifest differently across languages and cultures. These warrant further investigation with larger, more culturally and linguistically diverse samples to better understand how these findings translate to children globally. Third, while phonological processing is a core deficit in dyslexia, relying solely on phonological awareness tests may not fully capture the complexity of reading challenges, as it represents just one aspect of reading ability. Further research, which takes broader cognitive measures like working memory and attention into account, will need to be undertaken. Fourth, significant differences in parental education and family income between groups were observed, which may have confounded the results concerning depressive symptoms and behavior problems (Horoz et al., 2022; Mikkonen et al., 2016; Noonan et al., 2018). However, due to limitations imposed by sample size, these socioeconomic variables were not controlled as covariates in our analysis to maintain statistical power. This constraint limits the interpretability of mental health outcomes, and future studies are encouraged to include larger, more varied samples and to explicitly incorporate socioeconomic factors in their statistical models. Finally, the reliance on self-report and parent-report instruments to assess depressive symptoms and behavior problems represents another limitation, as these measures can be susceptible to reporting bias and subjective interpretation. Future studies might benefit from incorporating objective or clinician-rated assessments to bolster the robustness of findings.

## 5. Conclusions

In summary, this study demonstrates the complex interactions among reading ability, depressive symptoms, and internalizing and externalizing behavior problems among Chinese school-age children, highlighting the significant psychological implications associated with dyslexia. Mediation analysis indicates that depressive symptoms and behavior problems not only mediate but also reciprocally interact with reading ability, creating a cyclical negative feedback loop that exacerbates both academic difficulties and mental health issues. Practically, these findings point out the importance of adopting a multidisciplinary approach that integrates educational and psychological interventions in the management of dyslexia. Schools and clinical practitioners should implement comprehensive screening programs to detect mental health issues early in children with dyslexia, facilitating timely and targeted interventions. However, the case–control design limits causal inferences, and the relatively small and culturally specific sample restricts the generalizability of these findings beyond Chinese school-age children. Future research using longitudinal designs and more diverse, extensive samples across various cultural and linguistic contexts will be essential to clarify the bidirectional relationships identified here and to develop effective, culturally adaptive interventions for children with dyslexia.

## Figures and Tables

**Table 1:** Summary of pre-specified mediation models.

Model	X (Predictor)	M (Mediator)	Y (Outcome)
A	Reading ability	Internalizing behavior problems	Depressive symptoms
B	Reading ability	Externalizing behavior problems	Depressive symptoms
C	Reading ability	Depressive symptoms	Internalizing behavior problems
D	Reading ability	Depressive symptoms	Externalizing behavior problems
E	Depressive symptoms	Internalizing behavior problems	Reading ability
F	Depressive symptoms	Externalizing behavior problems	Reading ability
G	Internalizing behavior problems	Depressive symptoms	Reading ability
H	Externalizing behavior problems	Depressive symptoms	Reading ability

**Table 2:** Demographic characteristics of participants.

Variables	Total (*n* = 125)	Dyslexic (*n* = 44)	Control (*n* = 81)	*p*
Age, Mean ± SD	9.33 ± 1.24	9.57 ± 1.21	9.20 ± 1.24	0.163
Sex, *n* (%)				
Boys	61 (48.8)	27 (61.4)	34 (42.0)	0.038
Girls	64 (51.2)	17 (38.6)	47 (58.0)	
Grade, *n* (%)				0.948
Grade 2	31 (24.8)	11 (25.0)	20 (24.7)	
Grade 3	29 (23.2)	9 (20.5)	20 (24.7)	
Grade 4	31 (24.8)	11 (25.0)	20 (24.7)	
Grade 5	34 (27.2)	13 (29.5)	21 (25.9)	
Father’s education level, *n* (%)				0.001
Junior high school or below	35 (28.0)	20 (45.5)	15 (18.5)	
High school	28 (22.4)	11 (25.0)	17 (21.0)	
College or above	62 (49.6)	13 (29.5)	49 (60.5)	
Mother’s educational level, *n* (%)				<0.001
Junior high school or below	34 (27.2)	22 (50.0)	12 (14.8)	
High school	34 (27.2)	10 (22.7)	24 (29.6)	
College or above	57 (45.6)	12 (27.3)	45 (55.6)	
Family income per month (CNY), *n* (%)				<0.001
<5000	16 (12.8)	14 (31.8)	2 (2.5)	
5000~	45 (36.0)	20 (45.5)	25 (30.9)	
≥10,000	64 (51.2)	10 (22.7)	54 (66.7)	

Notes: CNY, Chinese Yuan, CNY 1 ≈ USD 0.15. *p* values were determined using Mann–Whitney *U* test for continuous variables and Chi-square test for categorical variables.

**Table 3:** Comparison of depressive symptoms between the dyslexic and control groups.

Variables	Dyslexic (*n* = 44), M (P_25_, P_75_)	Control (*n* = 81), M (P_25_, P_75_)	χ^2^/*Z*	*p*
Depression, *n* (%)	18 (40.9)	14 (17.3)	8.355	0.004
Scale scores	14.00 (10.25, 18.00)	8.00 (4.50, 12.00)	4.440	<0.001
Low Positive Affect	11.00 (6.25, 13.00)	6.00 (3.00, 10.00)	3.358	<0.001
Negative Affect	4.00 (1.25, 7.00)	1.00 (0.00, 3.00)	4.399	<0.001

Notes: M, Median. P_25_, P_75_, the 25th and 75th percentiles. *p* values were calculated by the Chi-square test for whether the child is depressed or not, and the Mann–Whitney *U* test was used for the scale scores of DSRS and two dimensions, while the Kolmogorov–Smirnov test indicated non-normal distributions (*Z*_DSRS scores_ = 0.084, *Z*_Low Positive Affect_ = 0.109, *Z*_Negative Affect_ = 0.217, all *p* < 0.05).

**Table 4:** Comparison of internalizing and externalizing behavior problems between the dyslexic and control groups.

Variables	Dyslexic (*n* = 44), M (P_25_, P_75_)	Control (*n* = 81), M (P_25_, P_75_)	*Z*	*p*
Internalizing behavior problems	8.00 (1.00, 14.00)	0.00 (0.00, 2.00)	5.274	<0.001
Externalizing behavior problems	5.00 (3.00, 9.50)	1.00 (0.00, 2.00)	5.866	<0.001

Notes: M, Median. P_25_, P_75_, the 25th and 75th percentiles. The Kolmogorov–Smirnov test indicated non-normal distributions for CBCL raw scores (*Z*_Internalizing_ = 0.260, *Z*_Externalizing_ = 0.277, both *p* < 0.001), leading to the use of Mann–Whitney *U* test for comparisons.

**Table 5:** Correlation analysis between variables (Spearman’s Rho).

	Mean ± SD	1	2	3	4
1. Reading ability	0.80 ± 0.22	-			
2. Depressive symptoms	10.74 ± 6.28	−0.31 **	-		
3. Internalizing behavior problems	3.56 ± 5.51	−0.39 **	0.34 **	-	
4. Externalizing behavior problems	3.91 ± 6.60	−0.44 **	0.35 **	0.77 **	-

Notes: *n* = 125. SD, Standard deviation. ** *p* < 0.01.

**Table 6:** Standardized mediation effects and 95% bootstrap confidence intervals.

Model Pathways	Indirect Effect			Direct Effect			Total Effect			Ratio of Indirect to Total Effect
	Effect	Boot LLCI	Boot ULCI	Effect	Boot LLCI	Boot ULCI	Effect	Boot LLCI	Boot ULCI	
A: RA → IBP → DS	−0.090 ***	−0.171	−0.026	−0.319 ***	−0.499	−0.138	−0.409 ***	−0.574	−0.243	22.03%
B: RA → EBP → DS	−0.119 ***	−0.231	−0.044	−0.290 **	−0.470	−0.110	−0.409 ***	−0.574	−0.243	29.12%
C: RA → DS → IBP	−0.083 ***	−0.165	−0.022	−0.358 ***	−0.535	−0.181	−0.441 ***	−0.605	−0.276	18.78%
D: RA → DS → EBP	−0.102 ***	−0.187	−0.035	−0.356 ***	−0.529	−0.184	−0.458 ***	−0.620	−0.296	22.23%
E: DS → IBP → RA	−0.114 ***	−0.193	−0.054	−0.290 ***	−0.455	−0.126	−0.405 ***	−0.568	−0.241	28.24%
F: DS → EBP → RA	−0.136 ***	−0.233	−0.066	−0.269 **	−0.436	−0.102	−0.405 ***	−0.568	−0.241	33.53%
G: IBP → DS → RA	−0.099 ***	−0.190	−0.036	−0.329 ***	−0.492	−0.166	−0.428 ***	−0.588	−0.268	23.12%
H: EBP → DS → RA	−0.105 ***	−0.202	−0.030	−0.345 ***	−0.511	−0.178	−0.450 ***	−0.608	−0.291	23.34%

Notes: RA, Reading ability. DS, Depressive symptoms. IBP, Internalizing behavior problems. EBP, Externalizing behavior problems. Boot LLCI, the lower limit of 95% bootstrap confidence interval. Boot ULCI, the upper limit of 95% bootstrap confidence interval. ** *p* < 0.01, *** *p* < 0.001.

**Table 7:** Regression analysis: the impact of reading ability on depressive symptoms.

Outcome Variable	Predictor Variable	*R*	*R* ^2^	*F*	*β*	*t*
Model A. Reading ability → Internalizing behavior problems → Depressive symptoms						
Depressive symptoms	Reading ability	0.45	0.20	10.04	−0.41	−4.90 ***
Internalizing behavior problems	Reading ability	0.45	0.21	10.44	−0.44	−5.30 ***
Depressive symptoms	Reading ability	0.48	0.23	9.08	−0.32	−3.50 ***
	Internalizing behavior problems				0.20	2.28 *
Model B. Reading ability → Externalizing behavior problems → Depressive symptoms						
Depressive symptoms	Reading ability	0.45	0.20	10.04	−0.41	−4.90 ***
Externalizing behavior problems	Reading ability	0.48	0.23	12.12	−0.46	−5.60 ***
Depressive symptoms	Reading ability	0.50	0.25	10.06	−0.29	−3.18 **
	Externalizing behavior problems				0.26	2.88 **

Notes: Sex and age were adjusted as covariates. All the variables were centralized and standardized. * *p* < 0.05, ** *p* < 0.01, *** *p* < 0.001.

**Table 8:** Regression analysis: the impact of reading ability on internalizing and externalizing behavior problems.

Outcome Variable	Predictor Variable	*R*	*R* ^2^	*F*	*β*	*t*
Model C. Reading ability → Depressive symptoms → Internalizing behavior problems						
Internalizing behavior problems	Reading ability	0.45	0.21	10.44	−0.44	−5.30 ***
Depressive symptoms	Reading ability	0.45	0.20	10.04	−0.41	−4.90 ***
Internalizing behavior problems	Reading ability	0.49	0.24	9.39	−0.36	−4.00 ***
	Depressive symptoms				0.20	2.28 *
Model D. Reading ability → Depressive symptoms → Externalizing behavior problems						
Externalizing behavior problems	Reading ability	0.48	0.23	12.12	−0.46	−5.60 ***
Depressive symptoms	Reading ability	0.45	0.20	10.04	−0.41	−4.90 ***
Externalizing behavior problems	Reading ability	0.53	0.28	11.71	−0.36	−4.10 ***
	Depressive symptoms				0.25	2.88 **

Notes: Sex and age were adjusted as covariates. All the variables were centralized and standardized. * *p* < 0.05, ** *p* < 0.01, *** *p* < 0.001.

**Table 9:** Regression analysis: the Impact of depressive symptoms on reading ability.

Outcome Variable	Predictor variable	*R*	*R* ^2^	*F*	*β*	*t*
Model E. Depressive symptoms → Internalizing behavior problems → Reading ability						
Reading ability	Depressive symptoms	0.46	0.21	10.53	−0.40	−4.90 ***
Internalizing behavior problems	Depressive symptoms	0.37	0.14	6.40	0.35	4.03 ***
Reading ability	Depressive symptoms	0.55	0.30	12.88	−0.29	−3.50 ***
	Internalizing behavior problems				−0.33	−4.00 ***
Model F. Depressive symptoms → Externalizing behavior problems → Reading ability						
Reading ability	Depressive symptoms	0.46	0.21	10.53	−0.40	−4.90 ***
Externalizing behavior problems	Depressive symptoms	0.42	0.18	8.86	0.39	4.68 ***
Reading ability	Depressive symptoms	0.55	0.30	13.13	−0.27	−3.18 **
	Externalizing behavior problems				−0.34	−4.10 ***

Notes: Sex and age were adjusted as covariates. All the variables were centralized and standardized. ** *p* < 0.01, *** *p* < 0.001.

**Table 10:** Regression analysis: the impact of internalizing and externalizing behavior problems on reading ability.

Outcome Variable	Predictor Variable	*R*	*R* ^2^	*F*	*β*	*t*
Model G. Internalizing behavior problems → Depressive symptoms → Reading ability						
Reading ability	Internalizing behavior problems	0.48	0.23	11.98	−0.43	−5.30 ***
Depressive symptoms	Internalizing behavior problems	0.39	0.15	7.35	0.34	4.03 ***
Reading ability	Internalizing behavior problems	0.55	0.30	12.88	−0.33	−4.00 ***
	Depressive symptoms				−0.29	−3.50 ***
Model H. Externalizing behavior problems → Depressive symptoms → Reading ability						
Reading ability	Externalizing behavior problems	0.50	0.25	13.14	−0.45	−5.60 ***
Depressive symptoms	Externalizing behavior problems	0.43	0.19	9.33	0.39	4.68 ***
Reading ability	Externalizing behavior problems	0.55	0.30	13.13	−0.34	−4.10 ***
	Depressive symptoms				−0.27	−3.18 **

Notes: Sex and age were adjusted as covariates. All the variables were centralized and standardized. ** *p* < 0.01, *** *p* < 0.001.

## Data Availability

The data used and/or analyzed during the current study are available from the corresponding author on reasonable request.

## Supplementary Materials

The following supporting information can be downloaded at: https://www.mdpi.com/article/10.3390/bs15081032/s1, Table S1. Correlation analysis between variables among boys (Spearman’s Rho). Table S2. Correlation analysis between variables among girls (Spearman’s Rho).
