# Supplementary material for: Reading Ability and Mental Health: Mediating Roles of Depressive Symptoms and Behavior Problems in Chinese School-Age Children
Source: Behav Sci (Basel). 2025 Jul 29;15(8):1032. doi: 10.3390/bs15081032 (PMC12382755; doi:10.3390/bs15081032)
Supplement: Supplementary file 1 [file behavsci-15-01032-s001.zip › behavsci-3748480-supplementary.pdf]

**Reading Ability and Mental Health: Mediating Roles of Depressive Symptoms and Behavior Problems in Chinese School-Age Children**

**Table S1** Correlation analysis between variables among boys (Spearman's Rho)

| | Mean ± SD | 1 | 2 | 3 | 4 |
|---|---|---|---|---|---|
| 1. Reading ability | 0.76 ± 0.24 | - | | | |
| 2. Depressive symptoms | 11.10 ± 6.88 | -0.37** | - | | |
| 3. Internalizing behavior problems | 3.43 ± 5.03 | -0.36** | 0.38** | - | |
| 4. Externalizing behavior problems | 4.87 ± 6.99 | -0.46** | 0.40** | 0.81** | - |

Notes: $n$ = 61. SD, Standard deviation. $^{**}p<0.01$.

**Table S2** Correlation analysis between variables among girls (Spearman's Rho)

| | Mean ± SD | 1 | 2 | 3 | 4 |
|---|---|---|---|---|---|
| 1. Reading ability | 0.84 ± 0.19 | - | | | |
| 2. Depressive symptoms | 10.41 ± 5.69 | -0.22 | - | | |
| 3. Internalizing behavior problems | 3.69 ± 5.97 | -0.46** | 0.30* | - | |
| 4. Externalizing behavior problems | 3.00 ± 6.12 | -0.43** | 0.31* | 0.80** | - |

Notes: $n$ = 64. SD, Standard deviation. $^{*}p<0.05$, $^{**}p<0.01$.
